# O8-5 Sociodemographic factors associated with discrepancy between body satisfaction change and weight change among school-aged adolescents following a behavioral intervention

**DOI:** 10.1093/eurpub/ckac094.061

**Published:** 2022-08-29

**Authors:** Florian Manneville, Francis Guillemin, Karine Legrand, Edith Lecomte, Jenny Ann Rydberg, Serge Briançon, Abdou Yacoubou Omorou

**Affiliations:** 1 APEMAC, Université de Lorraine, Vandoeuvre-Lès-Nancy, France; 2 CIC-1433 Clinical Epidemiology, CHRU de Nancy, Vandoeuvre-Lès-Nancy, France; 3 ISTNA, CNAM, Nancy, France

**Keywords:** discrepancy, body satisfaction change, weight change, youth

## Abstract

**Background:**

Interventions promoting healthy behaviors such as physical activity are effective to prevent overweight and obesity among adolescents. Following such interventions, body satisfaction change could be discrepant with weight change (e.g. less body satisfaction while having lost weight), and decrease sustainability of behaviors in the long-term. This study aimed to describe the discrepancy between body satisfaction change and weight change among adolescents following a 2-year school-based intervention, and to identify associated sociodemographic factors.

**Methods:**

Adolescents from the 2-year school-based ‘Promotion de l'Alimentation et de l'Activité Physique’ study conducted in northeastern France from 2006 to 2009 were included. Body satisfaction change was assessed using a self-administered questionnaire at the end of the study. Weight change was measured by the difference of body mass index z-score at end and start of the study. Discrepancy between body satisfaction change and weight change was described with cross-tabulations and weighted Cohen's kappa. Sociodemographic factors associated with discrepancy were determined by multivariate logistic regression models.

**Results:**

Among the 3279 adolescents included (mean ± standard deviation age= 15.2±0.6 years), the proportion of discrepancy between body satisfaction change and weight change was 74.8% (pessimism= 41.6%; optimism= 33.2%). The weighted Cohen's kappa indicated high discrepancy (?= 0.09; 95% confidence interval [0.07; 0.11]). The likelihood of discrepancy, especially pessimism was higher in boys than in girls (odds ratio= 1.44, 95% confidence interval [1.19; 1.74], p= .0002), and higher in adolescents with high socioeconomic status than in those with low socioeconomic status (odds ratio= 1.82; 95% confidence interval [1.20; 2.74], p= .004)

**Conclusions:**

Discrepancy between body satisfaction change and weight change was high among school-aged adolescents with increased likelihood for boys and adolescents with high socioeconomic status. Body satisfaction change should be considered in overweight and obesity prevention interventions alongside body weight change, and could be used as an indicator of long-term behavior maintenance.

